# Fighting while Parasitized: Can Nematode Infections Affect the Outcome of Staged Combat in Beetles?

**DOI:** 10.1371/journal.pone.0121614

**Published:** 2015-04-01

**Authors:** David Vasquez, Anna Willoughby, Andrew K. Davis

**Affiliations:** 1 Department of Biological Sciences, Virginia Polytechnic Institute and State University, Blacksburg, Virginia, 24061, United States of America; 2 Department of Evolutionary Anthropology, Duke University, Durham, North Carolina, 27708, United States of America; 3 Odum School of Ecology, University of Georgia, Athens, Georgia, 30602, United States of America; Universidade Federal de Minas Gerais, BRAZIL

## Abstract

The effects of non-lethal parasites may be felt most strongly when hosts engage in intense, energy-demanding behaviors. One such behavior is fighting with conspecifics, which is common among territorial animals, including many beetle species. We examined the effects of parasites on the fighting ability of a saproxylic beetle, the horned passalus (*Odontotaenius disjunctus*, Family: Passalidae), which is host to a non-lethal nematode, *Chondronema passali*. We pitted pairs of randomly-chosen (but equally-weighted) beetles against each other in a small arena and determined the winner and aggression level of fights. Then we examined beetles for the presence, and severity of nematode infections. There was a non-significant tendency (p = 0.065) for the frequency of wins, losses and draws to differ between beetles with and without *C*. *passali*; non-parasitized individuals (n = 104) won 47% of their fights while those with the parasite (n = 88) won 34%, a 13% difference in wins. The number of nematodes in a beetle affected the outcome of fights between infected and uninfected individuals in an unexpected fashion: fighting ability was lowest in beetles with the lowest (p = 0.033), not highest (p = 0.266), nematode burdens. Within-fight aggression was highest when both beetles were uninfected and lowest when both were infected (p = 0.034). Collectively, these results suggest the nematode parasite, *C*. *passali*, is associated with a modest reduction in fighting ability in horned passalus beetles, consistent with the idea that parasitized beetles have lower energy available for fighting. This study adds to a small but growing body of evidence showing how parasites negatively influence fighting behavior in animals.

## INTRODUCTION

By definition, parasites subsist off of the resources of their host, in many cases draining these resources and reducing host performance or fitness [1–5]. The majority of work in this area has been focused on the effects of parasites on host development or growth of immature stages [6], or on the reproductive output of adults [7, 8]. The effects of parasites on behavior are less well studied. Many animals must secure and defend resources or territories by fighting conspecifics in energetically costly contests. Individuals that are infected with parasites may be at a disadvantage in these situations. Indeed, while some work on this subject has shown parasites have minimal effects on fighting ability [9], most studies have found that parasites reduce their host’s fighting ability or competitiveness [10–12], compared to non-parasitized individuals. These studies have been conducted on crickets infected with tachinid flies [11], trout infected with trematodes [10] and salamanders with ectoparasitic mites [12]. In the current study, we examined the effects of a naturally-occurring nematode parasite on the fighting ability of a saproxylic beetle.

**Fig 1:**
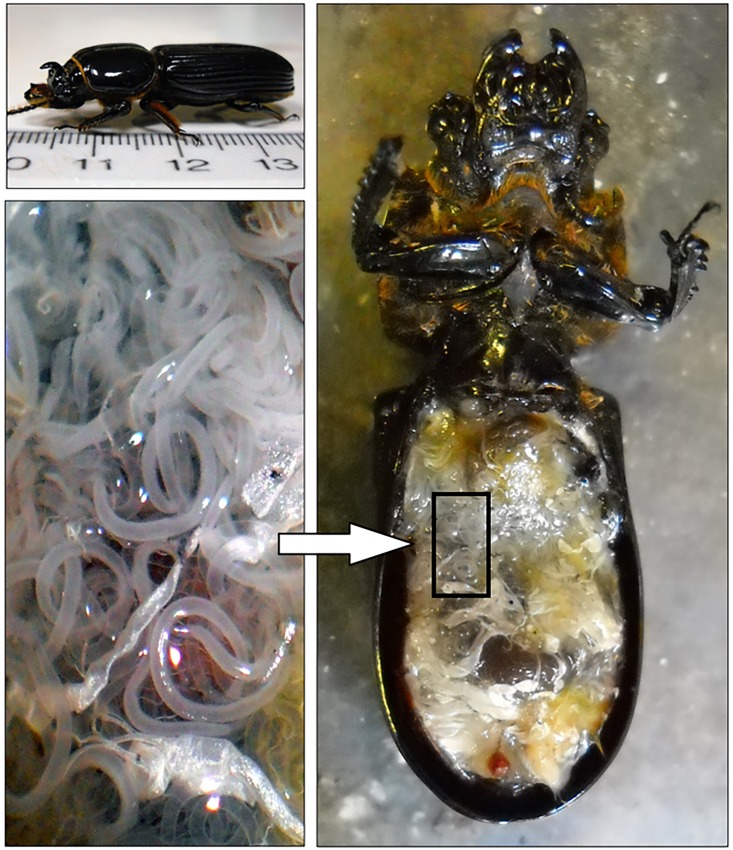
Live horned passalus beetle (*Odontotaenius disjunctus*, top left) and a freshly-killed specimen (right) with a heavy nematode infection (*Chondronema passali*, bottom left). Infections of this nematode can involve thousands of individual worms.

Our study subject was the horned passalus (*Odontotaenius disjunctus*, Fig. 1), which is a medium-sized (1–2 g) beetle native to the United States. Sometimes called ‘betsy beetles,’ or ‘bess beetles,’ they are found in decaying hardwood logs on the forest floor and their range includes most of the eastern seaboard. The beetles live in galleries or burrows they excavate in the logs, and burrows usually contain 1–2 adults plus 3–6 larvae in the summer. As their name suggests, they (males and females) have a small decurved horn on the top of their head, with female horns being slightly larger (relative to their body size) than male horns [13]. On average, female beetles are about 12% larger overall than males [14]. Intraspecific fighting occurs in passalus beetles when individuals defend their burrow against intruding beetles attempting to take over the burrow [14–16]. Both males and females defend the burrows [16]. Fights in passalid beetles can be easily reproduced in the laboratory, and the behaviors associated with fighting in this species (and related passalids) have been well-documented [16–18]. Generally, these behaviors are consistent with those of other beetles, including biting, grappling, or attempting to lift and/or flip the opponent [19–21]. Flipping behavior of this beetle can be seen in S1 Video.

The horned passalus is also host to a wide variety of external and internal parasites, including mites [22], tachinid flies [23, 24], and two species of nematodes. One of these (*Histrignathus rigidus*) lives entirely in the gut in low abundance, while the other, *Chondronema passali*, occurs in the abdominal cavity at varying levels of abundance, sometimes numbering in the thousands [22, 25, 26]. In fact, one report indicated a single beetle had over 4000 individual *C*. *passali* worms [22]. Thus, while the horned passalus is host to a range of parasites, none would have as much influence on the host biology as this nematode. This species of nematode is not well-studied, and its taxonomic status is unclear [27], although it is known to be prevalent in most beetles collected (i.e. 60–100%) throughout their range in the United States [22, 25, 26, 28], including the location of our project (north Georgia). Given this high prevalence combined with the sometimes high severity, it stands to reason this parasite is non-lethal. Nevertheless, this parasite could have substantial sub-lethal effects on hosts, especially for severe infections; a single nematode weighs approximately 0.015mg (Davis, unpubl. data), so infections of 1000 worms would make up 1% of the body weight of a beetle weighing 1.5g. Empirical studies on the effect of this parasite on passalus beetles are rare; one study attempted to determine if the parasite resulted in reductions in physical performance in this beetle, but found no overall negative effect [26]. Interestingly, the same study also found that wild-caught beetles parasitized with *C*. *passali* tended to be larger in size than non-parasitized individuals. The reason for this last pattern is unclear, but may be related to parasite-induced increases in feeding rates in larvae (investigations are currently underway in our lab). Both of these results are counter-intuitive given the extreme parasite burdens, and possible energy drain of this nematode on its host.

Here we performed an experiment aimed at determining if the *C*. *passali* nematode affects the fighting ability of its host, the horned passalus. Our approach was to stage combats between pairs of individual beetles collected in the wild and selected at random and then determine the parasite status of each beetle. The primary goal was to determine if beetles that are infected with nematodes win fewer fights than do uninfected beetles. We also determined whether infected beetles are more or less aggressive and have less stamina during fights. If parasite infections reduce energy available for fighting, we predicted that infected beetles should lose more often than uninfected. Moreover, fights where both beetles are uninfected should tend to be more aggressive than those where both are infected. Finally, parasite infections may lead to reduced stamina, or shorter fights in general.

## METHODS

### Beetle collection and housing

Beetles for this project were collected from rotting hardwood logs in forested areas surrounding Athens, GA USA (33.95°N, 83.38°W) during June and July 2014. All collecting was done on property owned by the University of Georgia, and no collection permits were required; the horned passalus is not a protected species in the United States. Logs were manually opened using hatchets and adult beetles were excavated and placed in plastic containers filled with wood debris for transport to the lab. Once in the lab, all beetles collected in each field trip were mixed together, then we randomly placed them in groups of 20 in 20L glass aquaria filled with hardwood pieces, until the beetles were used in fighting trials (below). The aquaria were misted daily to keep the wood moist. We made multiple collections of beetles during these months to obtain the number of beetles required for the project (n = 192), but importantly, we always ensured that beetle pairs consisted of individuals from the same collection trip so that their handling and housing experience prior to fights was similar.

### Arena setup

**Fig 2:**
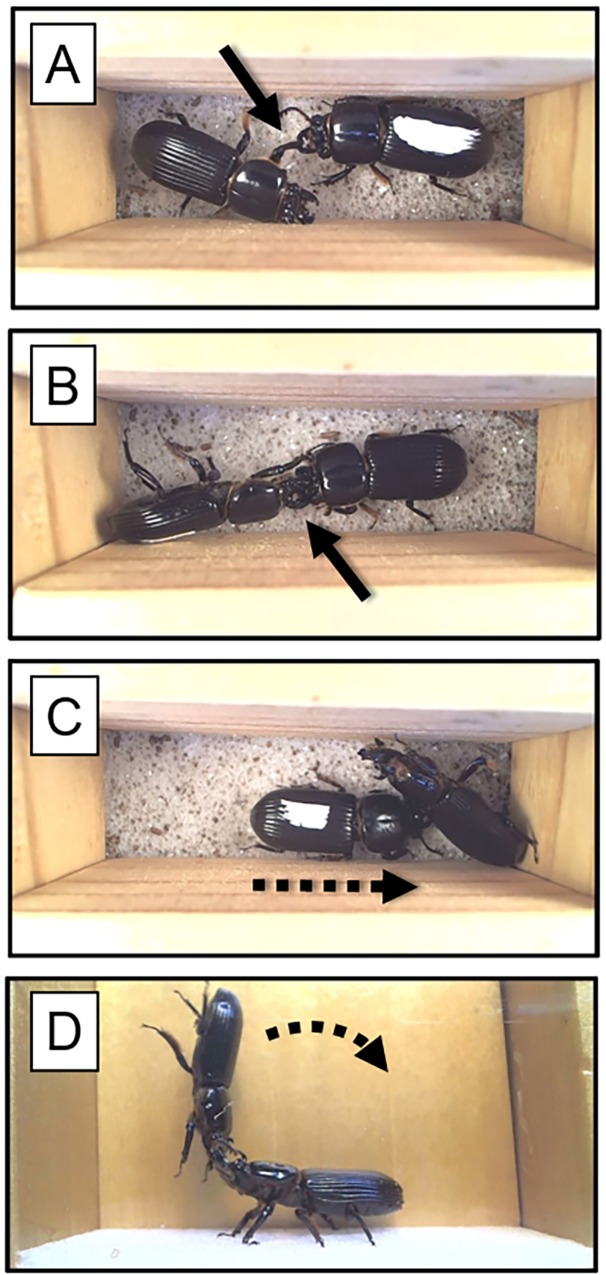
Still photos from the video-recording of beetle fighting. The arena was made of glued wood pieces and was 8cm long, 2.4cm wide and 3.5cm deep. A foam pad on the floor provided grip to the beetles. Panels A and B show biting behavior (black arrow). Panel C shows one beetle crawling under the other. Panel D shows a side image from an arena with one glass side, which we made to film one beetle flipping the other (shown). Note that these behaviors can be seen in S1 and S2 Videos.

We created a simple arena made of glued wooden blocks in order to watch and record beetle fights. The arena was 8cm long, 2.4cm wide and 3.5cm deep; these dimensions allowed room for two beetles, but it restricted their movement except toward each other (Fig. 2). This can be considered a ‘forced-fight’ setup, since it did not allow the individuals to withdraw from each other [11]. We note that in the wild these beetles live in wood galleries that tend to be smaller (narrower) than this (Davis, *pers*. *observations*), although fights in passalid beetles can also occur outside the gallery entrance [18]. The arena floor was made of hard foam to provide grip for the beetles. We secured a close-focus video camera on a stand just above the arena that was connected to a computer. The video frame encompassed the entire arena so that both beetles were visible at all times and all behaviors could be monitored (see S2 Video).

### Recording fights

Beetles were randomly assigned to fighting pairs based only on their weight (g), so that each pair consisted of beetles that weighed approximately the same (within 5% of each other). This was to eliminate the effect of body size, which is known to affect fight outcomes in most beetles [29, 30, 31], and to isolate the effects, if any, of nematode infections. We used body weight as an index of size; preliminary work showed weight is highly-correlated with total body length (n = 21 beetles, r = 0.90, p<0.00001; Davis, *unpubl*. *data*). Note that we had no knowledge of nematode infection status prior to fights, nor did we know the gender of the beetles until their dissection later. One beetle in each pair was marked with a dab of white-out on its elytra, then the beetles were placed in the arena facing each other. Once released, the beetles usually commenced fighting, or initiated aggressive behaviors. If they did not (i.e. if after 20 seconds there was no interaction), they were removed from the arena briefly, and re-positioned again inside. We found that most fights could be elicited after one or two such attempts. The fights were recorded for 3 minutes, which we determined to be an appropriate timeframe in preliminary trials. During the trials one observer (DV) monitored all bouts, and when each bout ended (i.e. one beetle retreated or assumed a submissive posture), the observer immediately re-positioned the beetles to stimulate a new bout. Thus, all beetle ‘fights’ were composed of multiple bouts within the 3 minute timeframe. For most pairs in this study there were between 8–10 bouts. We elected to study beetle fights in this manner because in our preliminary trials we noticed that sometimes a beetle will win one bout first but lose the next 6 or 7, so that multiple bouts would be needed to determine the true winner. This approach, of recording multiple bouts in a single session, has been utilized in behavioral studies of other species of insects and invertebrates [32–34]. Moreover, the timeframe of fights we used (3 minutes) is consistent with the behavior of passalid beetles, where fights can last up to 4 minutes and contain more than one bout [18]. A sample fighting video, containing 3 minutes of bouts, is shown in S2 Video.

### Assessment of fights

One of us (AW), who had no knowledge of beetle infection status, reviewed the fighting videos. For each bout the observer recorded the time of the start and end of the bout (based on the video timestamp), and assigned the bout winner and loser, based on known aggressive (bites, flips, etc.) or submissive (i.e. retreating) behaviors in these beetles (see results, Tables 1 and 2). A bout was considered a draw when there was no movement or other behavior for ~20 seconds, and the observer had to reset the beetles. A full list of behaviors we observed in this study is shown in Table 1. The overall winner was the beetle with the most wins. Draws were recorded when both beetles won equal numbers of bouts. In addition, the observer assigned an ‘aggression score’ for each bout which ranged from 1–5 based on the number of aggressive behaviors observed (by either beetle) in the bout: 1 = no movement; 2 = one beetle exhibited aggression, no biting; 3 = both beetles exhibited aggression, no biting; 4 = both beetles were aggressive and bit each other; and 5 = one beetle flipped the other. The overall average score was then determined for each pair. Similar fight-intensity scoring systems have been employed to study staged combat in crickets [11]. Finally, the observer recorded the final behavior that decided the outcome of each bout, such as a bite, or a flip (see results). These behaviors were obvious as they usually led to the losing beetle retreating.

**Table 1:** Ethogram of behaviors observed in passalus beetles in this study.

**BEHAVIOR**	**DESCRIPTION**
**Approach**	Beetle walks toward opponent
**Investigate**	Stationary beetles move antennae at each other with no contact
**Pause**	Beetle retracts antennae and is stationary
**Move Away**	Beetle retracts from opponent; Beetle leaves or attempts to leave arena
**Head Contact**	Beetle's mandibles are in contact with opponent, no aggressive movements
**Other Contact**	Beetle's elytra or legs are in contact with opponent’s
**Climb Under/Over**	Beetle forcefully walks over/under opponent
**Bite**	Beetle closes mandible on opponent
**Flip**	Beetle lifts opponent vertically with mandible
**Non-physical**	Beetle retreats after no approach or contact

**Table 2:** Summary of behaviors that led to the ending of fighting bouts, listed in order of frequency of occurrence. This list encompasses all bouts from the 96 beetle pairs (n = 787 bouts).

**Behavior Category**	#** Observations**	%** of total**
**One beetle crawls over the other**	13	1.7
**One beetle approaches, other retreats**	29	3.7
**One beetle flips the other**	69	8.8
**One beetle crawls under the other**	95	12.1
**Non-physical end** *	119	15.1
**One beetle bites the other**	150	19.1
**No activity, bout terminated by observer**	312	39.6
**Grand Total (bouts)**	**787**	**100**

*Both beetles retreat without contact or other activity

### Nematode infection

At the end of each fight, we killed the beetles in ethanol, and immediately dissected open their abdominal cavity to determine nematode infection status and beetle gender (based on the presence or absence of the male aedeagus). Because we could not determine infection status of beetles prior to fights, in some pairs, both beetles were uninfected, or both were infected, or only one was. The nematode (*C*. *passali*) typically is found throughout the abdominal cavity and is easily observed with a low-power dissecting microscope, especially when the beetle is freshly-killed (Fig. 1). We are confident in the identification of this nematode; this beetle is host to only two nematode species, and *C*. *passali* is the only worm parasite present in the abdominal cavity [22, 28]. Finally, we scored the intensity of parasite infection on a 0–3 scale, with 1 being less than 10 worms, 2 being less than 100 and 3 being 100–1000+.

### Data analyses

The primary variable of interest in this study was the frequency of fights won by beetles (n = 192), and we wanted to know if this was affected by parasite infection (and severity). Thus we compiled the number of fights won (i.e. number of overall wins, not the number of bouts) and lost by all uninfected beetles, and did the same for beetles in each nematode infection category (1–3). The frequency of wins, losses and draws for each beetle group was then compared using Chi-square tests. Note that this approach utilizes data from both beetles in each pair, which may be a pseudoreplication issue. However, we found that the overall results of these comparisons did not change when we used one randomly-chosen beetle from each pair (n = 96, Davis, unpubl. data), thus we report results using the full data set. We also compared frequencies of wins, losses and draws between males and females, and found no significant difference (χ^2^ = 0.652, df = 2, p = 0.722).

Next, we examined certain components of actual fights to determine effects of nematode infection on fighting intensity and stamina. For this we capitalized on the groupings that resulted from the random selection of beetles: both beetles uninfected, only one infected, and both infected. We used ANOVA to compare 1) the aggression score of fights, 2) the percentage of fights that ended in flips (the most energetically-intensive behavior), and 3) the average bout time (in seconds) of beetles across these three groups. Tests in this study were performed using the STATISTICA 12.1 software package (StatSoft, Tulsa, USA).

## RESULTS

### General

Within the 96 beetle fights we examined in this study, there were a total of 787 fighting bouts. Bouts lasted an average of 13.8 seconds (range 7–28 seconds). We observed a range of aggressive and passive behaviors, listed in Table 1. The aggressive behaviors that ended fights are listed in Table 2, in reverse order of occurrence. The most common fight-ending behavior was when one beetle bit the other (19% of fights; Fig. 2A,B). Crawling over or under the opponent (Fig. 2C) occurred about 14% of the time, while flipping the opponent (Fig. 2D) was observed about 9% of the time. Combined, these 3 aggressive behaviors accounted for 41.7% of all cases.

### Effects of parasitism

Beetles that were not parasitized with *C*. *passali* (n = 104) were deemed the overall winner in 47.1% of their fights (Table 3), winning 49, losing 42 and with 13 draws. Meanwhile, beetles with nematodes (all groups) won 34.1% of their fights, with frequencies of wins, losses and draws being 30, 37 and 21, respectively (Table 3). These frequencies were not statistically different, although the test statistic approached significance (χ^2^ = 5.47, df = 2, p = 0.065). The largest difference appeared to be in the frequency of draws, which occurred in 23.9% of fights of nematode-parasitized beetles compared to 12.5% in non-parasitized beetles (Table 3). The severity of nematode infection of a beetle appeared to affect whether it won a fight. Beetles with the lowest severity score (category 1, or less than 10 nematodes) only won 19.0% of their fights (compared to 47.1% in non-parasitized beetles), and their fights ended in draws 28.6% of the time (compared to 12.5% in uninfected; Table 3). These frequencies were significantly different (χ^2^ = 6.81, df = 2, p = 0.033). Beetles with the moderate infection score (category 2, or between 10 and 100 worms) won a similar proportion of their fights (46.9%) as did non-parasitized individuals, but they lost fewer (25.0%) and had more draws (28.1%). These differences approached significance (χ^2^ = 5.27, df = 2, p = 0.072). Finally, beetles with the highest nematode burdens (100 to 1000+) won 31.4% of their fights, lost 51.4% of the time, and had draws in 17.1% of cases. These frequencies were not significantly different from those of uninfected beetles (χ^2^ = 2.65, df = 2, p = 0.266). Collectively, these results indicate that the largest effect of nematodes on fight outcome appears to be confined to those beetles with low nematode burdens; these beetles either won fewer fights and/or had more contests ending in draws as compared to beetles without nematodes.

**Table 3:** Outcomes of all fights from the 96 beetle pairs examined in this study (192 beetles), grouped by nematode infection score. In each pair the winning beetle was the individual that won the most bouts in the 3 minute timeframe. When both beetles won equal numbers of bouts the fight was considered a draw.

**Nematode Score**	#** Wins**	#** Losses**	#** Draws**	**Total Beetles**
**0 (no worms)**	49 (47.1%)	42 (40.4%)	13 (12.5%)	104
**1 (<10 worms)**	4 (19.0%)	11 (52.4%)	6 (28.6%)	21
**2 (10–100 worms)**	15 (46.9%)	8 (25.0%)	9 (28.1%)	32
**3 (100–1000+ worms)**	11 (31.4%)	18 (51.4%)	6 (17.1%)	35
**All infected**	30 (34.1%)	37 (42.0%)	21 (23.9%)	88
**Grand Total**	79 (41.1%)	79 (41.1%)	34 (17.7%)	192

**Fig 3:**
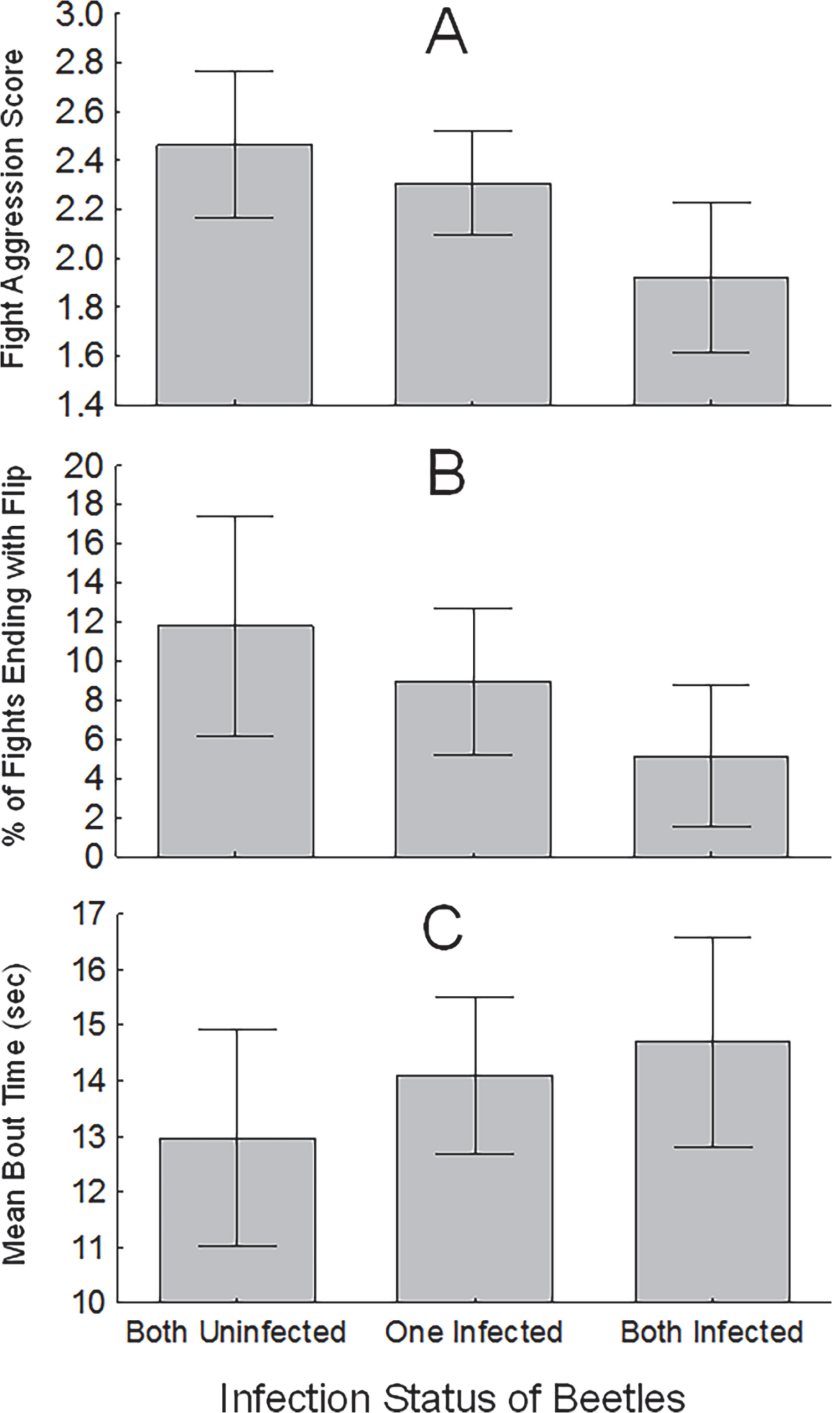
Charts showing the relative intensity and stamina of beetle fights according to the infection status of the pair. Chart A shows the average aggression score of fights, which was a 1–5 scale assigned by the video observer (who had no knowledge of beetle infection status), based on the number of aggressive behaviors observed per bout (see ethogram in Table 1 for behavior descriptions). Chart B shows the percentage of fights that ended with one beetle flipping the other (the most aggressive behavior). Chart C shows the average time per fighting bout for each infection category.

When the fight aggression scores were compared across the three categories of beetle pairings (both beetles uninfected, one infected, and both infected), we found significant variation (F_2,93_ = 3.49, p = 0.034), with highest average aggression scores in the pairs where both beetles were uninfected and lowest in those where both were infected (Fig. 3A). The same analysis that examined the percentage of fights ending with one beetle flipping the other yielded no significant difference between groups (F_2,93_ = 1.73, p = 0.182), although we note that the pattern of these means (Fig. 3B) is similar to the aggression scores; when both beetles were infected the mean percentage of time the fights ended with a flip was 11.8% (± 14.7 SD), compared to 5.1% (± 7.9 SD) when both were infected. Results of these two analyses suggest a negative effect of nematode parasites on energy expended during fights. Finally, the analysis of bout duration indicated there was no difference across fight groupings (F_2,93_ = 0.89, p = 0.414; Fig. 3C), but we do note that the trend was for infected beetle pairings to have longer bouts than those pairs with no parasites, which was counter to our prediction.

## DISCUSSION

Our results provide evidence that *Chondronema passali* nematode infections are associated with a modest reduction in overall fighting ability in the horned passalus; beetles with these nematodes tended to win less often (the effect approached significance), and fights between two infected beetles tended to be less intense, with fewer high-energy behaviors. Surprisingly, the largest effect was seen in the beetles with the lowest parasite burdens, while those with heavy infections appeared to be less affected (Table 3). This pattern seems counter-intuitive; one would expect the largest energy drain (and by extension, the largest effect on fighting) to be in the beetles with the highest, not lowest, parasite burdens. One explanation for this pattern may be that beetles with heavy infections could be older (i.e. infections have had longer to build in older beetles), and presumably these older individuals would be more experienced at fighting [35]. In other words, age and infection could be confounded, preventing us from detecting the true effect of infection severity (on fighting ability). Passalus beetles can live over 2 years [36], though it is not possible to determine age in field-collected passalus beetles, so we have no way of testing this idea with our data. A secondary explanation relates to the residual reproductive value hypothesis, which reasons the less likely an animal is to live to the next unit of time the more likely they are to allocate resources now to a costly behavior (in this case, fighting) [37–39]. Put another way, if heavily-infected beetles are not likely to survive long (which we do not know), they may invest more into brood defense (i.e. fighting) than would a lightly-infected beetle, which may have a higher chance of survival. This could explain our results concerning fighting ability of heavily-infected beetles, although to be sure of this explanation, further experiments examining parasite-effects on survival would be needed.

The fact that heavily-parasitized individuals appeared just as effective in winning fights as non-parasitized beetles was intriguing in itself; it is not immediately clear how beetles with such heavy nematode burdens can function at such a high level throughout this (or in any) behavior. Future work would be needed to determine the physiological effects of such heavy infections, and this could involve examining hemolymph to determine concentrations of lipid, protein and/or carbohydrate levels [8, 40, 41], which may give clues to the available energy.

While our predictions concerning fight outcome were generally supported, we found no evidence that the nematode parasites reduce stamina in passalus beetles, as we had predicted. In fact, the average length of fighting bouts of two infected beetles tended to be slightly longer than that of two uninfected beetles (Fig. 3C). This could be explained by the higher intensity level of the latter case (Fig. 3A); when both beetles were uninfected, their fights tended to be decided sooner because of the higher level of aggression, while in the opposite case, the parasitized beetles more often engaged in less-intense, non-physical behaviors that are inherently longer in duration (approaching, investigating, pausing). Given that we only recorded 3 minutes of bouts, our fighting setup may not have allowed us to determine stamina in the beetles. On the other hand, the timeframe we used is not unrealistic in terms of the biology of passalid beetles; prior work demonstrated their territorial fights rarely last more than 4 minutes in laboratory settings [18]. Given the short timeframe of fights in this species, physical stamina may not be relevant.

We note that the fighting behavior we observed in this study generally matched what has been reported in prior studies of this beetle [16, 17] and related passalids [18]. The most extreme behavior—grabbing and lifting or flipping the opponent—was less often used compared to other forms of aggression (biting, crawling under), which may reflect its energetic cost. Other studies have also found this behavior is infrequent [16]. This behavior was difficult for the beetles to perform in our study because the opponent was usually holding tight to the substrate (i.e. resisting the flip). We know the weight of the beetles is not a factor in the flipping behavior; prior work showed these 2g beetles can lift over 500g [42]. Parasitized beetles tended to refrain from this behavior (Fig. 3B), which is consistent with the idea that it requires a high degree of energy.

While the collective results of this project point to parasite-induced reductions in fighting ability, it should be noted that the magnitude of the effect was small; parasitized beetles won 34% of their fights while non-parasitized individuals won 47% (Table 3), which is only a 13% difference. This may make sense given the relative ubiquity of the nematode; it appears to be present in 60–100% of beetles in most collections [14, 22, 25–27] (46% in our study). If these parasites did cause more dramatic reductions in fighting behavior, over time this would effectively remove parasitized individuals from the breeding population, since they would tend to be displaced by non-parasitized beetles in their burrows. Further, since this behavior usually results in the intruder killing the larvae of the burrow resident [43], this would eliminate any parasites in the larvae as well. Larvae are known to harbor the parasite, although prevalence in that stage is closer to 20% [28]. All of this would result in low parasite abundance within populations, which is not what is observed. Thus, the relatively small impact of nematodes may be the result of selective pressure on the parasite for reduced virulence on important host fitness traits (such as fighting).

To be fair, the small effect of nematodes on fighting ability, combined with the unexpected pattern in infection severity (beetles with the lowest infections suffered most), could be interpreted as evidence that this parasite does not really affect fighting in passalus beetles. This idea seems plausible since prior evidence showed this parasite does not impact physical strength of the host [26]. However, strength was measured in that project in a passive state (i.e. beetles were not manipulated or stressed), which may not mimic the intense energy demand of physical combat. Moreover, beetles pulled a force gauge, and this pulling strength may not be as biologically relevant as lifting strength, which would be important during physical contests in beetles, where lifting the opponent is a key to victory [19–21]. Indeed, recent work in our lab showed passalus beetles are capable of lifting much more than they can pull, and that their lifting strength doubles when in a stressed state [42]. Most importantly, preliminary work using lifting strength data has shown this nematode parasite does reduce the amount of weight beetles can lift (Davis, unpubl. data), and this would logically translate into reduced fighting success.

Beetles are frequently the subjects in studies of fighting behavior, especially with respect to their often-elaborate horns or other weapons [19, 21, 44–46]. However despite the many studies on the subject, to our knowledge, ours is the first study of beetle fighting that examined the possible effects of parasites. Given that our results did show moderate detrimental effects, it would be appropriate to encourage other researchers to examine their beetle subjects for parasites following fighting trials (regardless of the goal of the study), since such information would help interpret patterns in the data.

In conclusion, our study provided evidence that the nematode parasite, *C*. *passali*, was associated with a reduction in fighting ability in horned passalus beetles (although the effect was not large), consistent with the idea that parasitized beetles have lower energy available for fighting. This conclusion is similar to those drawn from studies of other host-parasite systems [10–12], which speaks to the general nature of the phenomenon. However, the finding that beetles with light infections (as opposed to severe) performed poorest was surprising, and warrants additional research. For the behavioral ecologist, this project also emphasizes the importance of considering the parasite burdens of test animals when interpreting results from staged contest trials.

## Supporting Information

S1 Data Excel file containing all fight data used for analyses. (XLSX)

S1 Video Flipping behavior of horned passalus beetles, one of the most aggressive behaviors during fights. One beetle grasps the other and lifts, presumably to dislodge the opponent from the log. Note that the beetle being flipped attempts to hold the substrate, and prevent being lifted. This would translate into a greater energy cost for the aggressor.
(MP4)

S2 Video A sample fighting video, containing 3 minutes of bouts between two passalus beetles. There were 6 distinct bouts in this video, starting at timestamps 0:10, 0:54, 1:53, 2:22, 2:44 and 2:57. The behavior that ended each bout was (in order): Flip, Flip, Non-physical, Bite, Flip and Fight termination by observer (see Table 2). The winning beetle of each bout was as follows: Black, Black, White, Black, White and Draw.
(WMV)
